# Examining the ability of palm kernel shell extract to control corrosion and assess its economic value on thermo-mechanically treated steel in artificial seawater: a sustainable and environmentally friendly approach

**DOI:** 10.3389/fchem.2024.1396565

**Published:** 2024-05-14

**Authors:** Omotayo Sanni, Jianwei Ren, Tien-Chien Jen

**Affiliations:** Department of Mechanical Engineering Science, University of Johannesburg, Cnr Kingsway and University Roads, Johannesburg, South Africa

**Keywords:** palm kernel shell, seawater inhibition, carbon steel corrosion, green corrosion inhibition, sustainability

## Abstract

Each year, the rising demand for palm oil generates large amounts of palm kernel shell waste. Discarded palm kernel shells can produce activated carbon, crushed shells, liquified fumes, and other derivatives; however, their indiscriminate disposal persists, raising issues related to the environment and economy. Therefore, the purpose of this study is to investigate the use of palm kernel shell as a corrosion inhibitor for thermo-mechanically treated steel in a seawater environment using gravimetric and electrochemical techniques, as well as surface tests at varying concentrations. The findings demonstrated that the palm kernel shell inhibited the cathodic and anodic processes by adsorption on the steel surface, which followed the Langmuir adsorption isotherm. The inhibitor exhibited a 98% inhibitory efficiency at 500 ppm concentration. Scanning electron microscopy analysis verified the thin films of the inhibitor on steel surface in seawater solution. Fourier transform infrared spectroscopy results show that the extract’s components prevent the steel corrosion through an adsorptive mechanism. According to the inhibitor economic evaluation, employing the palm kernel shell extract is less expensive than utilizing conventional inhibitors.

## 1 Introduction

Corrosion is a dangerous issue that can cause machine parts to fail and even cause deadly accidents. Impurities such as H_2_S, CO_2_, naphthenic acids, and chlorides can accelerate the corrosive action of metals and equipment utilized in the transportation, production, exploration, and refining of petroleum. Many steel and reinforced concrete constructions have been subjected to chloride ions, which has caused corrosion and reduced their longevity. Naidu and Joseph (2022) state that corrosion in reinforced concrete happens when an aggressive substance, such as chloride, diffuses into the reinforcement. Although there are several ways to prevent corrosion, using inhibitors is still the most popular method (Saha et al., 2015b; Saha et al., 2015a; Saha et al., 2016; Saha et al., 2019; Saha et al., 2021; Fouda et al., 2022; Saha et al., 2022; Wang et al., 2022; Bijapur et al., 2023; Li et al., 2023). Prior research has extensively examined the application of synthetic organic compounds with heteroatoms, heterocyclic rings, and aromatic rings to reduce corrosion aggression on steel. The majority of these artificial organic chemicals are hazardous and detrimental to the environment, despite the fact that they have a strong inhibitory effect. Thus, stringent environmental laws and heightened ecological consciousness among scientists have prompted the use of environmentally acceptable substitutes to aid in the prevention of corrosion on metal surfaces (Wei et al., 2020; Panchal et al., 2021; Verma et al., 2024). These naturally occurring compounds include molecules that have demonstrated a good inhibitory impact on metal corrosion, including polyphenols, amino acids, glucosinolates, alkaloids, and tannins. The current problem in the field of corrosion inhibition is the need to substitute these naturally occurring chemicals for the synthetic organic inhibitors. Extracts from our immediate surroundings are therefore needed for research, especially those that we consider to be waste.

Increased socioeconomic and infrastructural development brought about by the world’s population growth will replace rural lifestyles with urban ones, accelerate the depletion of natural resources, and produce large amounts of municipal solid waste. As a result, massive wastes are produced, the disposal of which presents several environmental issues. The majority of these wastes are biomass, which comes from food production and other agricultural processing operations. It is economically feasible to use these leftover components from the expanded agricultural operations because they are abundant and reasonably priced in large quantity (Dey et al., 2021). Fruit peels, hulls, shells, seed pods, bagasse, stalks, leaves, barks, roots, sludges, cobs, pulp, straws, brans, and husks are among the agricultural waste materials that are produced (Dey et al., 2021; Koul et al., 2022). The oil palm tree, which is grown in tropical regions of Asia, Africa, South America, and the Caribbean, produces the most amount of these waste materials in the form of fibers from the tree’s various parts (Descals et al., 2020; Qaim et al., 2020). Large amounts of palm kernel shells are produced during the extraction of palm oil from palm fruits, and these shells must be used for a variety of purposes. Research is currently being conducted on a variety of topics, such as the use of palm kernel shells as an additive in drilling fluids, livestock feed, construction aggregates, reinforcement for metals and polymeric composites, wastewater detoxifier, abrasive in automotive components, and bioenergy production (Safian et al., 2023; Borah and Das, 2022; Azunna, 2019; Mahlia et al., 2019; Saad et al., 2021; Razavi Mehr et al., 2019). Thus, effective waste management is essential for improving environmental conservation and profitably utilizing the wastes. While there has been a considerable amount of research on palm kernel shell, it has mostly concentrated on specific areas, and there is currently little information available regarding its usage as a corrosion inhibitor in various aggressive environments. Therefore, the purpose of this research is to solve disposal issues while also examining palm kernel shell extract (PKSE) inhibitive propensity as a value-added precursor to environmentally sustainable byproducts.

There are few reports in the literature on the use of palm kernel shell extract for corrosion protection. Earlier, we have reported the corrosion inhibition effect of palm kernel shell extract for stainless steel in 0.5 M H_2_SO_4_ solution with optimum efficiency of 93% (Sanni et al., 2022). The primary component of palm kernel shell has been detailed in the work of (Uchegbulam et al., 2022). Additionally, the use of palm kernel shell as corrosion inhibitor for stainless steel in simulated seawater medium has been reported with 90% inhibition efficiency (Sanni et al., 2021). To the best of our knowledge, no report on the use of palm kernel shell as corrosion inhibitor for Thermo-mechanically treated steel in seawater environment has been reported in the literature. In an attempt to extend the investigation of palm kernel shell extract for other engineering materials in different aggressive solutions, the present study is intended to report palm kernel shell waste aimed to alleviate the environmental pollution by converting waste into useful products, and to evaluate the inhibition action of palm kernel shell as a cheap and safe corrosion inhibitor for thermo-mechanically treated steel in corrosive media.

As previously said, the potential of palm kernel shell makes it clear that, even if it is a processing waste, there are alternative uses for it. Therefore, the goal of the current study is to turn waste into wealth. This study examines the effectiveness of palm kernel shell extract in a seawater media as a corrosion inhibitor for thermo-mechanical steel. Thermo-mechanically treated steel as a material of choice is a medium carbon steel that is primarily used by the construction industry as reinforcement in concrete structures (Ghosh and Ghosh, 2018). Seawater is chemically aggressive, and therefore, constructional materials used in seawater handling and processing systems are subjected to corrosion, hence seawater is the corrosive media used in this study. The current inhibitor was selected based on its low cost, lack of toxicity, lack of environmental concern, and ease of availability. This data will add to the body of knowledge that will help scientists make wise choices in the continuous effort to protect the environment by reducing carbon emissions and advancing greener energy sources.

## 2 Materials and methods

### 2.1 Preparation and extraction of palm kernel shell

The source of the palm kernel shell was Guateng, South Africa. The shell was air dried, after using a soft brush to remove debris, the shell was crushed. The method outlined by (Sanni et al., 2021; Sanni et al., 2022) was used to extract the crushed palm kernel shell. Using a rotary evaporator, the resultant extract was concentrated at 45°C in a vacuum. The property of the seawater was listed in Table 1.

**TABLE 1:** Properties of the seawater.

Compound	Amount (mg/L)
Sodium	10.510
Chloride	18.980
Sulfate	3.022
Calcium	3.80
Magnesium	1.627
pH	8.15
Conductivity	17.90 μs/cm
Salinity	10.22%
Alkalinity	11.80

### 2.2 Preparation of steel samples

The following are the chemical weight composition of the thermo-mechanically treated steel employed in this study: Fe (98.9%), C (0.13%), Si (0.11%), Mn (0.56%), S (0.03%), P (0.03%), Cu (0.10%), and Cr (0.06%). For weight loss study, the steel was cut into 2 cm × 0.5 cm × 2 cm, and for electrochemical studies, the samples had dimensions of 1 cm × 1 cm. To remove the surface oxides, the samples were mechanically abraded with emery sheets of varying grits.

### 2.3 Gravimetric measurements

The ASTM G31-7 standard procedure was followed to prepare 2 cm × 0.5 cm × 2 cm steel samples for the gravimetric test, the test solution was agitated to ensure homogeneity. The corresponding corrosive solutions (seawater medium, which acted as the blank, seawater with 100, 200, 300, 400, and 500 ppm concentration of the palm kernel shell extract) were submerged in 250 mL of each sample. For every experiment, fresh solutions under the identical conditions were employed. The specimen was mechanically polished using different grades of emery paper (180, 220, 360, 400, 600, 800, 1,000, and 1,200) prior to each submerging test. It was then washed and allowed to air dry. The corroded samples were removed at the end of each experiment, rinsed with distilled water, dried in acetone, and then reweighed. Eqs 1, 2 (Sanni and Popoola, 2019) were utilized to ascertain the inhibitory efficiency and corrosion rate of the palm kernel shell extract, respectively. The average weight loss readings were recorded and used to calculate the corrosion rate (CR in mm/year):

$$CR=\frac{87.6W}{DAT}$$ (1)

Where, W, A, T, and D represented the average weight loss (mg), the area of specimen (cm^2^), exposure time (h) and the density of metallic samples (g/cm^3^), respectively.

The inhibition efficiency was assessed from the corrosion rate based on Eq. 2:

$$IE=\frac{CRuninhibited-CRinhibited}{CRuninhibited}$$ (2)

To assess the impact of temperature on the corrosion inhibition of the palm kernel shell on steel substrates, the weight loss and corrosion rate of the steel at various temperatures (303, 313, 323, 333, and 343 K) were recorded in seawater medium with and without the inhibitor at varying concentrations.

### 2.4 Electrochemical measurements

Ag/AgCl and platinum rod served as the reference and counter electrodes, while the prepared steel, with 1 cm^2^ exposed area, served as the working electrode. Before the potentiodynamic polarization test, the system (electrodes, test solution) underwent a 30-min stabilization phase. To obtain insight into the corrosion mechanism and inhibition of the steel, a working electrode polarized from −0.25 to +0.25 mV was used, with a scan rate of 0.1 mV/s. The corrosion current density and corrosion rate were estimated from the polarization curve. Every test was run three times to guarantee the data’s accuracy and reproducibility.

### 2.5 Surface analysis

After 15 days of immersion in the corrosive solution, the surface of the sample with and without inhibitor was examined using a JEOL JSM-7600F scanning electron microscope running at an accelerating voltage of 15 kV. Energy dispersive X-ray spectroscopy was used to determine the elemental composition of the sample.

### 2.6 Economic evaluation

The direct cost of producing 1,000 g of the corrosion inhibitor under study in a laboratory setting was assessed. As a result, the energy costs associated with milling, drying, and screening as well as the transportation costs of the residue removal at the facility was taken into account (Carvalho et al., 2022), $ 0.09 was used. The average market value of commercial inhibitors was given in the work of (Carvalho et al., 2022) and these data are displayed in Table 2. Furthermore, our work aims to encourage the readjustment of a thriving corrosion inhibitor sector.

**TABLE 2:** The average price of commercial corrosion inhibitors (Carvalho et al., 2022).

Compound	Average price (*USD kg* ^−1^ *)*
Pyrazolone	150 ± 34
Benzothiazole	192 ± 44
Benzotriazole	195 ± 46
Benzethonium chloride	333 ± 44
Dibenzylthiourea	1930 ± 150

## 3 Results and discussion

### 3.1 Agro-industrial waste characterization

#### 3.1.1 Fourier transform infrared spectroscopy (FTIR)

**FIGURE 1:**
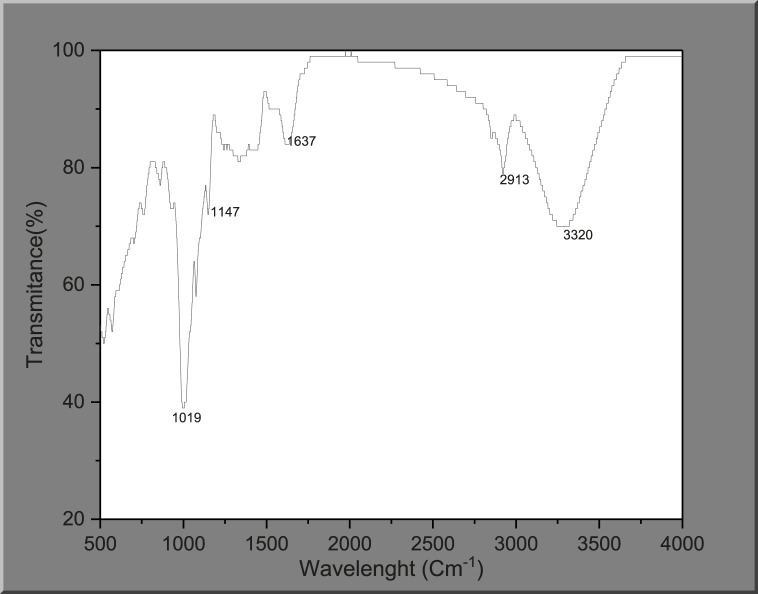
FTIR spectrum of palm kernel shell extract.

The absorption bands in FTIR spectra can be used to identify the behavior and performance of materials. An FTIR experiment was conducted to obtain an understanding of the components of the palm kernel shell extract. The palm kernel shell extract’s FTIR spectrum is displayed in Figure 1. It is clear from the FTIR data in Figure 1 that the PKSE contains several peaks, indicating more functional groups. The PKSE’s biodegradability is attributed to a peak that is remarkably visible in the 3,320 cm^−1^ wavelength. This peak is the stretched vibration from hydroxyl ion (–OH) as a result of absorbed moisture (Saber et al., 2019; Ikubanni et al., 2020). The absorption bands that stand out the most are 2,913, 1,637, and 1,147 cm^−1^. The distinctive alkyl groups are represented by the absorption band at 2,913 cm^−1^ (–COOH). The C=C is attributed to the absorption band at 1,637 cm^−1^. A flavonoid is associated with the absorption band at 1,147 cm^−1^. The distinctive adsorption of the glycosidic structure is represented by the bands that occur at 1,019 cm^−1^. Given the variety of phytochemical compounds present in the palm kernel shell, it is possible that some of the other phytochemical components will also chelate with ferric ions to guarantee robust adsorption on the steel surface. Therefore, the ability of the palm kernel shell to suppress corrosion may be the result of multiple elements working together. As a result, the FTIR result verifies that the extract under study has atoms and groups that can operate as adsorption centers, which is a crucial component of a corrosion inhibitor.

#### 3.1.2 Scanning electron microscopy (SEM)

**FIGURE 2:**
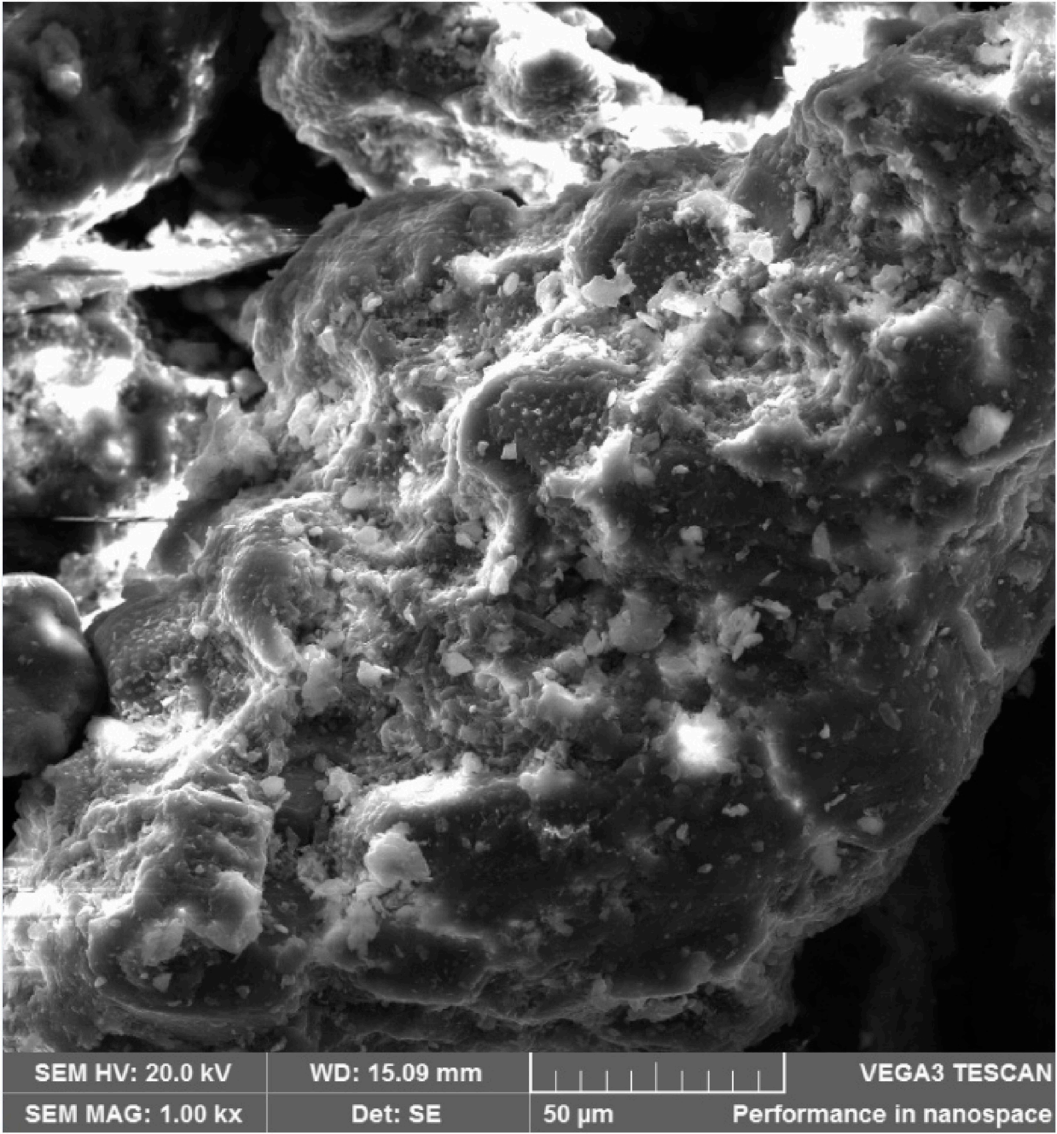
SEM image of PKSE agro-industrial waste.

**FIGURE 3:**
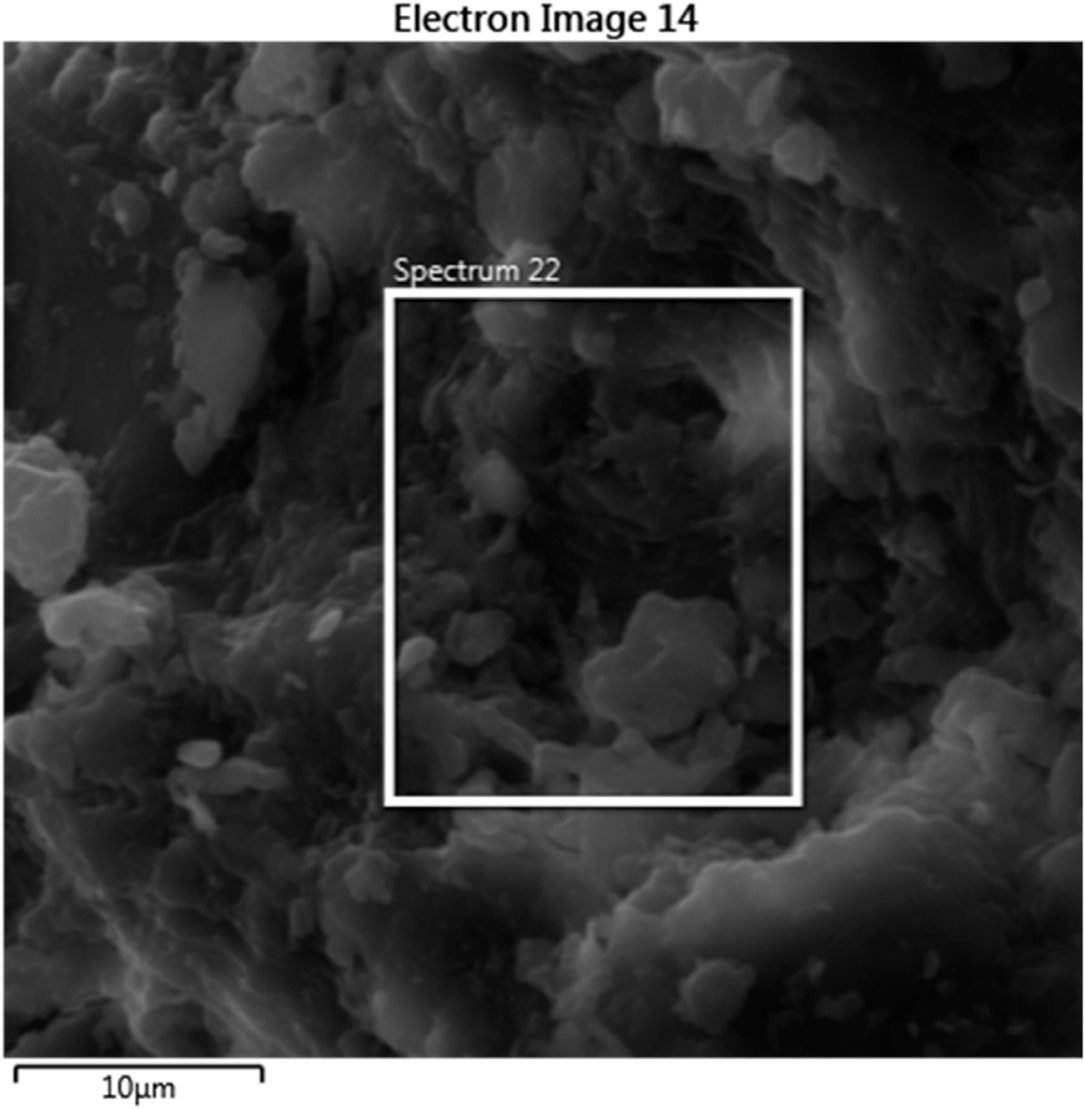
EDX mapping of PKSE agro-industrial waste.

The PKSE’s morphology was investigated by SEM imaging. As seen in Figure 2, the PKSE morphology is usually fibrous, alveolar, and exhibits uneven rods as a result of milling. In Figure 3, the EDX mapping is displayed.

It can be observed from Figure 3; Table 3 that the PKSE sample exhibits Kα1 lines at intensities to silicon, oxygen, and carbon. These components are distributed as follows: 0.2%, 29.2%, and 70.3% in that order. The chemical component of the PKSE as determined by elemental analysis is shown in Table 3. It is possible to conclude from the data in Figure 2 that the waste is primarily composed of organic components, such as C, O, Si, Fe, and Al. The organic molecules in the waste contain C and O, which may provide electron pairs for the waste to adsorb onto the metallic surface. This would lead to a partial blockage of the metal and, as a result, limit corrosion in the aggressive solution (Thakur and Kumar, 2021). It is feasible to suggest that this waste belongs to the class of inhibitory compounds because it contains both C and O atoms.

**TABLE 3:** Elemental analysis of the palm kernel shell extract.

Element	Wt%
C	70.3
O	29.2
Si	0.2
Fe	0.1
Al	0.1

#### 3.1.3 X-ray diffraction (XRD) analysis

**FIGURE 4:**
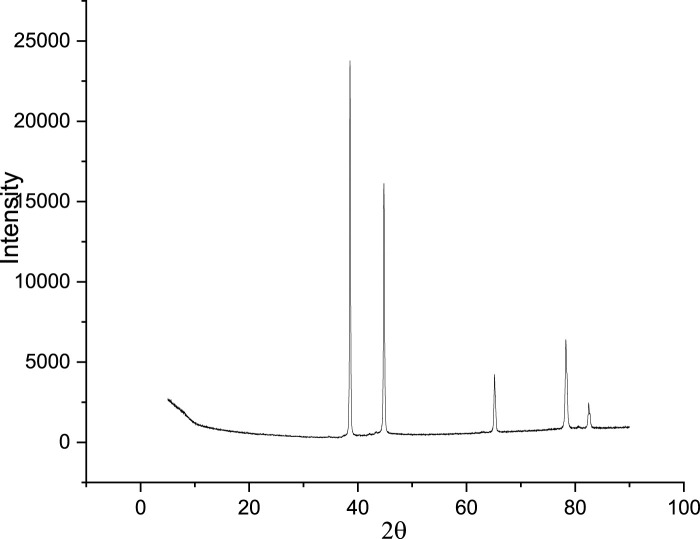
PKSE XRD analysis.

A foundational understanding of PKSE’s chemical configuration is provided by the compositional analysis. The XRD crystallinity analysis of PKSE is shown in Figure 4. The presence of carbon and oxygen atoms was revealed by the PKSE XRD patterns, with peaks at 2θ = 38 and 44, respectively. The PKSE is amorphous, as indicated by further peaks at 2θ = 65, 78, and 82. This is in line with findings from other research (Ikubanni et al., 2020; Jabarullah et al., 2021).

#### 3.1.4 X-ray fluorescence (XRF) analysis

XRF is an analytical technique that determines the presence and proportion of elements in a substance in order to determine its chemical composition. Table 4 shows that the main component in PKSE is silica, which is followed by phosphoric anhydride, quicklime, alumina, and magnesia. Because of the possible pozzolanic properties implied by the high silica, magnesia, and alumina levels, ash derived from PKSE has been investigated as a partial replacement for cement in cementations composites. Moreover, agricultural waste has the potential to be a corrosion inhibitor due to its availability, affordability, and other characteristics.

**TABLE 4:** Percentage chemical composition of PKSE from XRF analysis.

Compound	Edoziuno et al. (2021)	Ikubanni et al. (2020)	Hussain et al. (2019)
SiO_2_	55.69	46.20	31.50
MgO	4.85	3.70	16.90
Al_2_O_3_	9.43	2.30	16.50
CaO	11.21	15.10	10.60
P_2_O_5_	2.39	6.00	7.27
SO_3_	0.67	—	2.30
Cl	—	—	1.75
K_2_O	9.71	21.20	1.40
Fe_2_O_3_	3.32	3.20	0.83
MnO	0.72	0.60	0.61
Cr_2_O_3_	0.25	—	—
Na_2_O	1.76	1.00	—
TiO_2_		0.43	—

### 3.2 Electrochemical studies

#### 3.2.1 Electrochemical impedance spectroscopy (EIS)

**FIGURE 5:**
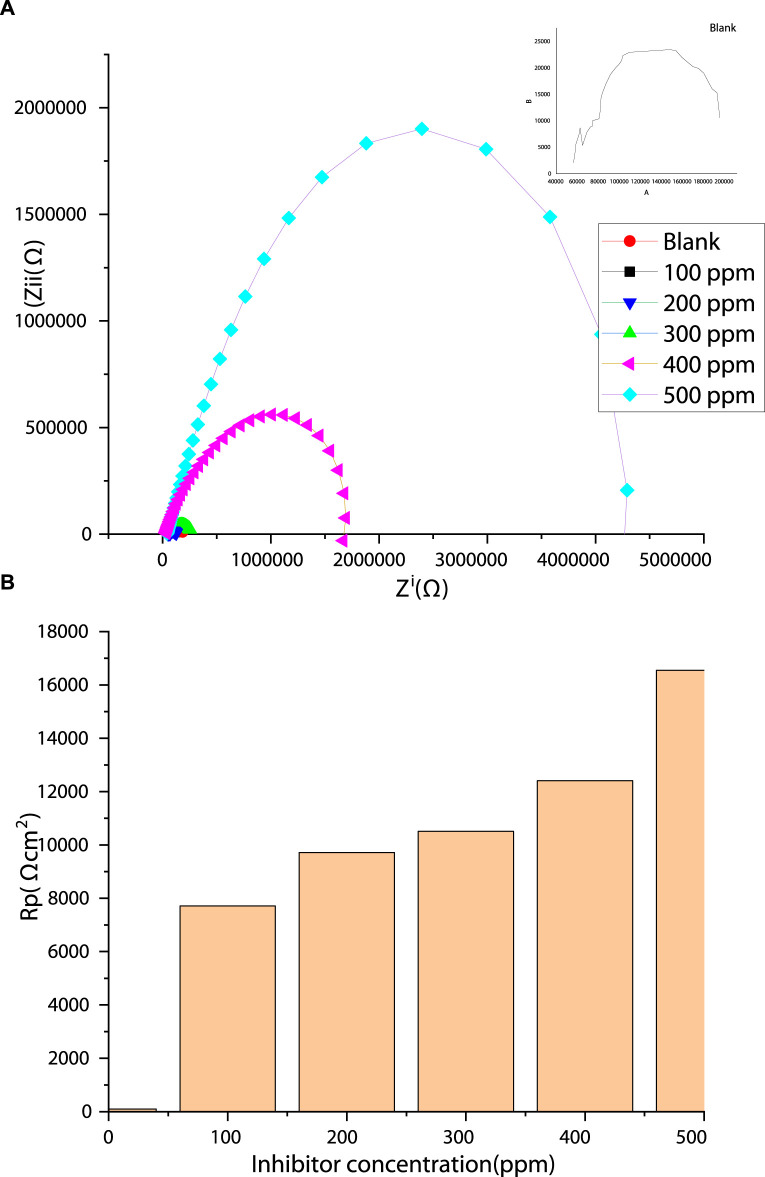
**(A)** Nyquist plots of the thermo-mechanically treated steel in saline solution in the presence and absence of the PKSE; **(B)** Rp with diverse PKSE concentration.

The impedance method was used to probe the steel/inhibitor interface and to characterize the adsorption and corrosion inhibition mechanism on the surface of the steel after the addition of PKSE extract. Impedance study provides a fast and reliable method for assessing the performance of corrosion inhibitor at metal/inhibitor interface. The advantage of impedance spectroscopy is that it can be used to measure the corrosion behavior of any inhibitor in a corrosive medium with respect to time and frequency. To support the electrochemical polarization experiments and to understand the formation and growth of the barrier film with increasing inhibitor concentration, an impedance study was performed. In line with several researches (Ozcan et al., 2004; Solmaz et al., 2008; Solmaz, 2010; Saha et al., 2015a; Saha et al., 2016), the corrosion inhibition potential of palm kernel shell extract was investigated. Figure 5 shows the Nyquist representations of the obtained results. Two phenomena are observed in the blank and the inhibited Nyquist spectra. In the spectrum of the blank, a single capacitive loop at high-to-medium frequencies and an inductive loop at low frequencies are observed. The capacitive loop, which is a common feature of a solid electrode, is due to a charge transfer phenomenon (Ozcan et al., 2004); that is, the corrosion of the steel in the aggressive solution is controlled by charge transfer (Ozcan et al., 2004; Solmaz et al., 2008; Solmaz, 2010). The inductive loop at the low frequencies is due to the relaxation of adsorbed corrosion products on the steel surface. The diameter of the semicircle increased with increasing inhibitor extract concentration, indicating an increase in charge-transfer resistance. The obtained Nyquist curves (Figure 5A) present a single semicircle capacitive loop for the entire frequencies, demonstrating that the added inhibitor did not alter the mechanism of corrosion (Solmaz, 2010; Saha et al., 2015a; Saha et al., 2016). In general, larger diameter of Nyquist semicircles indicates effective resistance against charge transfer process occurring at the interface of the metal/solution, signifying the lower corrosion rates. Noticeably, the semicircle’s diameters increased with the adding PKSE, demonstrating that the corrosion extent of the steel in the saline solution is reduced. At different concentrations of PKSE, the shapes of the Nyquist curves were not different in comparison with the blank saline over the investigated frequency region, signifying that the addition of PKSE produces a thin inhibitor film rather than altering the corrosion mechanism. The effect, however, is concentration dependent, and the 500 ppm concentration exhibits the maximum effect.

**FIGURE 6:**
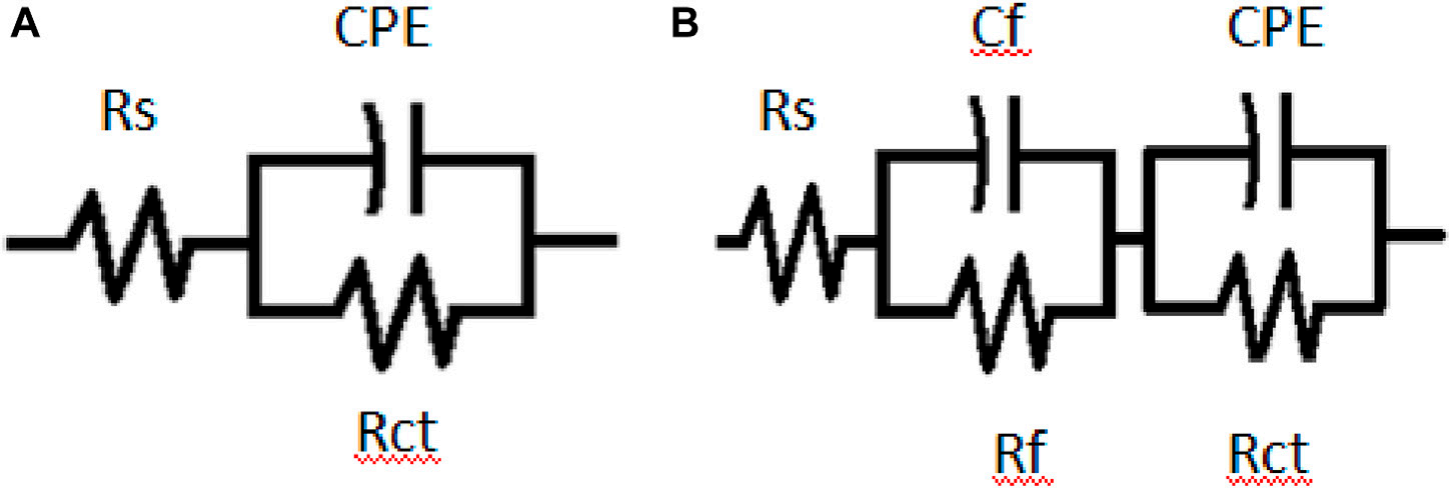
Equivalent circuits **(A)** blank and **(B)** with PKSE used to fit EIS data.

The equivalent circuit model in Figure 6 was utilized for EIS fitting analysis. The equivalent circuit in Figure 6A consists of a solution resistance (*R*s), a charge transfer resistance (*R*ct), and a constant phase element (CPE). In Figure 6B, the inductor is substituted with a second constant phase element and the inductive resistance with film resistance (*R*f). The resistance values increase as the extract’s concentration increases (Figure 5B), denoting higher resistance of the steel as a result of adsorption of the extract, possibly due to the interactions between the free doublets of the heteroatoms existing in PKSE molecules and the vacant d-orbitals of iron. From Figure 6, the 500 ppm concentration of the extract has the best performance, in other words, the waste can be used as a substitute for synthetic organic compounds for corrosive control.

#### 3.2.2 Potentiodynamic polarization studies

**FIGURE 7:**
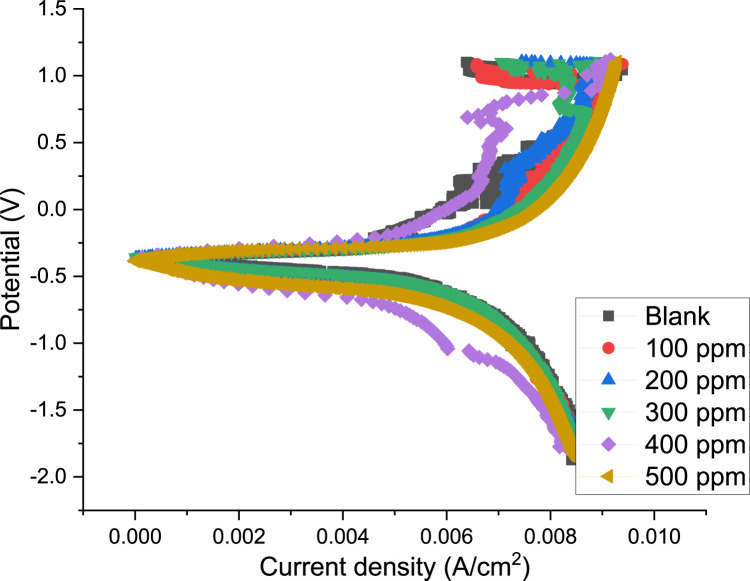
Polarization curves obtained for the steel in seawater in the presence and absence of the PKSE extract.

The steel’s anodic and cathodic corrosion responses were studied using the potentiodynamic polarization approach. Figure 7 shows the potentiodynamic polarization curves for the steel in blank and with various PKSE extract concentrations. Table 5 lists the polarization parameters, which are obtained by extrapolating the linear segments of the anodic and cathodic branches. These parameters include the corrosion potential (Ecorr), the corrosion current density (icorr), the cathodic Tafel slope (βc), and the anodic Tafel slope (βa). Eqs 3, 4, respectively, were used to determine the corrosion rate and the extract’s inhibitory efficiency.

$$CR=0.00327\frac{icorrxw}{d}$$ (3)

$$IE=\left(1-\frac{icorr}{icorr_{0}}\right)x100$$ (4)

**TABLE 5:** Electrochemical parameters obtained by potentiodynamic polarization of steel in seawater solution in the presence and absence of the PKSE extract.

Inhibitor concentration (ppm)	βa (V/dec)	βc (V/dec)	E*corr* (V)	*Icorr* (A/cm^2^)	Polarization resistance (Ω)	Corrosion rate (mm/year)
Blank	0.0821	0.5611	−0.3789	0.0095	16.83	2.3586
100	0.0656	0.0909	−0.3572	0.000123	101.85	0.9623
200	0.0532	0.0358	−0.3261	0.00021	808.03	0.1108
300	0.0228	0.1100	−0.3111	2.65E-05	877.47	0.0217
400	0.0785	0.4442	−0.1146	1.22E-05	2,552.61	0.0017
500	0.0747	0.1369	−0.2185	1.01E-06	3,011.20	0.0004

Where w is the equivalent weight of the substrate, icorr is the corrosion current density for the inhibited systems, icorr_0_ is the corrosion current density in the absence of inhibitor, and 0.00327 is a constant used for dimension and time conversion.

**FIGURE 8:**
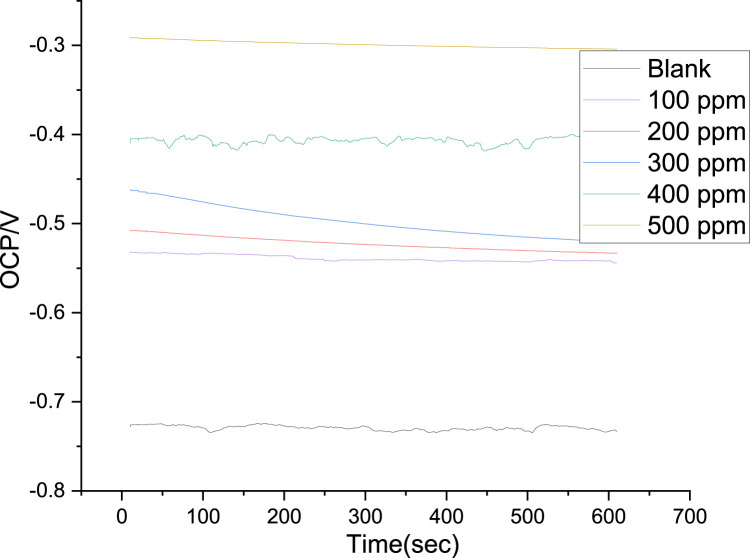
Open circuit potential variation of the steel in saline solution, with and without PKSE at different concentrations.

The cathodic region promoted the hydrogen evolution reaction and consequent removal of the oxide layer formed during the OCP stabilization. OCP in the control solution, evolution of the open circuit potential is characterized essentially by a slow decay to approximately 0.72 V (Figure 8), which is slightly above the reversible potential for the oxidation from Fe to Fe^2+^ This potential drop can be related to the dissolution of the native oxides and thus to the activation of the metal. The development of a thin layer on the metal surface caused the current density to abruptly fall. A drop in cathodic currents and a shift in the initial potential to more positive values were seen by the addition of the PKSE extract. The extract reduced the steel sensitization process, as evidenced by the active region’s decreased current densities when the inhibitor was present. Moreover, it was observed that the inhibitory effect on the steel surface was manifested by blocking active sites without changing the anodic and cathodic reaction mechanisms, forming a protective film on the metal surface, as evidenced by the reduction in current density in the inhibited solution for both cathodic and anodic Tafel branches (Solmaz et al., 2023). A protective layer that prevents ions from passing through the electric double layer and reaching the metal surface may be formed, as the corrosion current is likewise lowered as the inhibitor concentration rises. It is therefore recommended that PKSE has good corrosion inhibitory capability. According to Table 5 data, adding the PKSE extract to the reaction medium caused the corrosion current density of the steel samples to decrease and their polarization resistance increase. This suggests that the addition of the extract inhibits the corrosion process, which is consistent with findings from other studies (Maestro et al., 2023; Vorobyova et al., 2023). As the amount of PKSE extract increased, there was a corresponding shift in the cathodic tafel slope. Furthermore, the inclusion of the extract resulted in very little decrease in the anodic coefficient. This behavior implies that the PKSE extract adsorbed onto the surface, which are a feature of mixed-type inhibitors, facilitate the inhibition by a blocking action (Vorobyova et al., 2023). The extract is a mixed type inhibitor that is adsorbed on the metal surface and blocks the corrosion process, as suggested by the decreased corrosion current density values in the presence of inhibitor without significantly altering corrosion potential. The literature states that both physisorption and chemisorption phenomena will be considered in the corrosion inhibition when there is no discernible difference in the tafel slopes (Ehiemere et al., 2023; Rabizadeh et al., 2023; Sofiane et al., 2023). From the polarization data, the PKSE had a maximum inhibitory efficiency of 96.71%, suggesting that natural chemicals can serve as a viable replacement for synthesized organic compounds.

### 3.3 Gravimetric measurements

**FIGURE 9:**
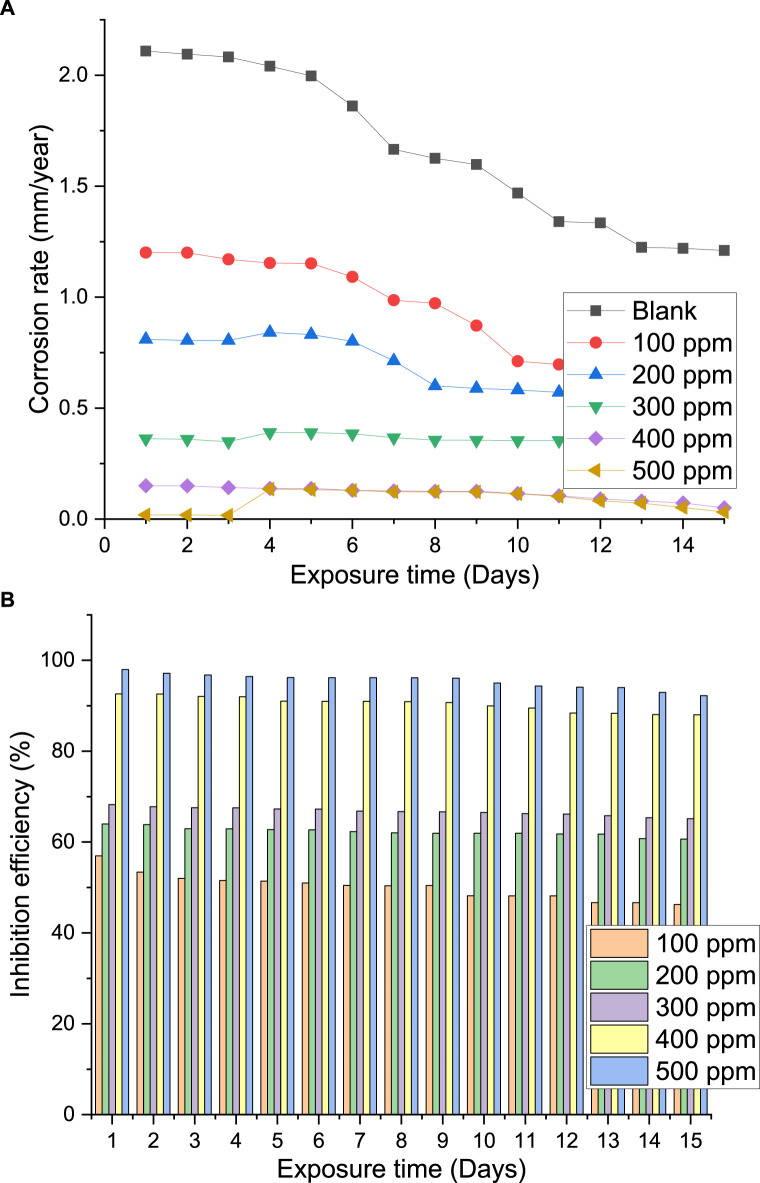
Plot of **(A)** corrosion rate of steel in seawater with and without palm kernel shell extract versus exposure time; **(B)** inhibition efficiency of palm kernel shell extract versus exposure time.

Because of its straightforward application and dependability, the weight loss method of evaluating the corrosion rate and inhibition efficiency is beneficial. Due to its ease of use, the weight-loss approach of monitoring corrosion and an inhibitor’s inhibitive efficiency has been used in various studies (Bijapur et al., 2023; Narang et al., 2023). To guarantee that the results were precise, each gravimetric experiment was carried out three times. The corrosion rate and inhibition efficacy against extract concentration based on the obtained results is shown in Figure 9. It is evident that when extract concentration rises, the corrosion rate decreases. Maximum extract adsorption and maximum corrosion mitigation occur at 500 ppm concentration. In a similar vein, an increase in inhibitive efficiency is noted with increasing extract concentration. Because it demonstrated the lowest corrosion rate and the maximum efficiency, 500 ppm is the optimal concentration of palm kernel shell extract for the steel in seawater solution.

#### 3.3.1 Effect of concentrations effect

Eq. 3 was utilized to compute the corrosion rate, based on the ASTM G31-72 standard (American Society for Testing and Materials) as previously documented (Bijapur et al., 2023; Narang et al., 2023). Figure 9A illustrates how the corrosion rate drops with concentration and reaches a peak inhibitory efficiency of 98% at 500 ppm. This shows that the extract effectively inhibits steel corrosion in seawater solutions, and even at low concentrations, it may be considered a good inhibitor. This result indicates that a protective coating can be applied to the exposed steel area since it shows good agreement between the two methodologies utilized in this investigation. Clearly aromatic compounds and heteroatoms, especially oxygen atoms, which operate as active centers and promote adsorption at the steel surface are included in the extracts under study. In fact, increasing inhibitor concentration directly affects how these naturally occurring compounds forms a protective layer on the steel surface, which lessens the influence of chloride. Adsorption of this chemical is facilitated by the synergistic action of non-binding electrons on the hetero-atoms that comprise the PKSE extracts and the presence of π-electrons in aromatic rings.

#### 3.3.2 Effect of immersion time

Figure 9 illustrates the impact of immersion time on the rate of corrosion and the inhibitory effectiveness of the palm kernel shell extract. The values in Figure 9 demonstrate how adding the extract reduced the pace at which steel corroded. The increase in inhibitory efficiency showed that greater extract concentrations promoted increased surface blocking. When the metal is being prevented from corroding, the solvent molecules are also adsorbed at the metal/solution interface. Regardless of the immersion time, the presence of inhibitors boosts the efficacy of inhibition. This could be as a result of the extracts’ phytochemical components adhering to the metal surface (Hossain et al., 2023). When compared to the corrosion rate without the inhibitor, the corrosion rate caused by the extract’s at varying concentrations was lower. Up until day 15, all concentrations showed an increasing trend in corrosion rate. Previous research indicates that the phenomena is caused by the protective layer on the steel surface desorbing as the immersion time increases and the adhesion strength between the protective film and the steel weakening (Cui et al., 2024). The inhibition efficacy with immersion time is displayed in Figure 9B. Because of the protective film’s adsorption, desorption, and re-adsorption on the steel, an upward and downward trend is seen. Furthermore, it is seen that the 500 ppm concentration of the extract generated greater efficiency in comparison to the other concentrations.

#### 3.3.3 Effect of temperature

**FIGURE 10:**
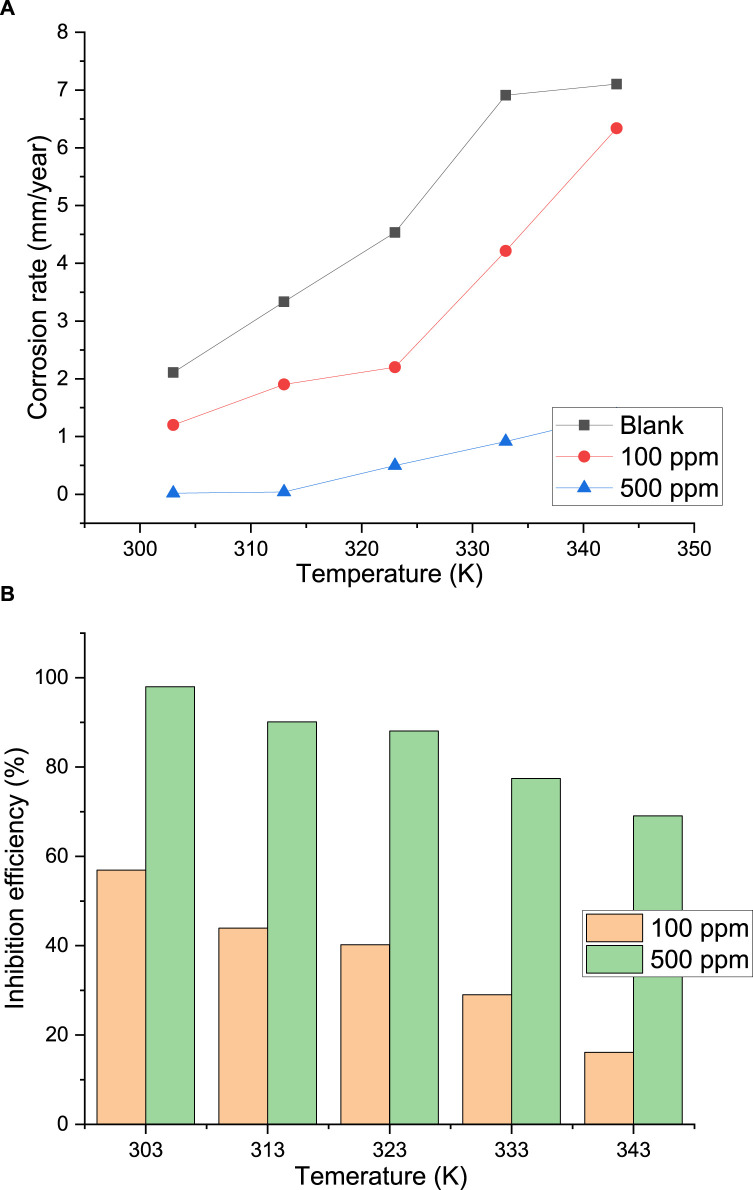
**(A)** Plot of corrosion rate of palm kernel shell extract against temperature, **(B)** efficiency of palm kernel shell extract against temperature.

One of the essential qualities of an effective corrosion inhibitor is its ability to retain a particular level of stability when used in a seawater environment at high temperatures. Furthermore, by comparing the activation apparent energies of the corrosion process with and without the presence of inhibitor in the aggressive media, this provides additional insight into the inhibitor action mechanism. Using the weight loss approach, the impact of temperature on the inhibitive effectiveness of palm kernel shell extract in seawater medium was evaluated at 303, 313, 323, 333, and 343 K. The findings are displayed in Figure 10. The addition of the extract decreased the corrosion rate of the steel for all temperatures, indicating that the extract adsorbs onto the steel surface and acts as an inhibitor in the seawater solution. In both the uninhibited and inhibited media, the corrosion rate of the steel increases with temperature and is caused by an increase in the kinetic energy of the corrosion reactions. The phytochemical components of the extract’s adsorption on the metal surface prevented corrosion reaction sites and slowed the pace of corrosion reactions, as evidenced by the fact that the corrosion rate is higher in the uncontrolled medium than in the inhibited medium. Thermic agitation, which encourages the adsorption–desorption instability of the inhibitory molecules at the metal surface, is probably the reason why the inhibitory efficiency of the PKSE declined with temperature (Yellezuome et al., 2022). This outcome validates the inhibitor’s desorption on the metal surface.

Upon meticulous examination of the impact of temperature on the inhibited systems, it can be observed that the inhibition efficiency rose (Figure 10B) and the corrosion rate decreased (Figure 10A). It is well recognized that the thickness, stability, and stiffness of an adsorption inhibitor film have a major impact on its effectiveness. One explanation for the observed low corrosion rates at lower temperature could be that, at low temperatures, a significant amount of the palm kernel shell extract molecules were more likely to adsorb on the steel surface, providing a reasonable level of protection (Figure 10A). One mechanism that may have characterized the adsorption of the extract molecules on the steel surface is chemical adsorption, as evidenced by the slight decrease in the rate of corrosion and the increase in the inhibitory efficiency of the PKSE extract upon temperature rise. Usually, the chemical adsorption process is consistent with an increase in inhibitory efficacy with system temperature. However, at higher temperatures (323–343 K), the equilibrium between adsorption and desorption may have shifted in favor of desorption, resulting in a shorter time lag between the extract molecules’ adsorption and desorption, which exposes the metal surface to the corrosive seawater solution for a longer amount of time. The observed higher rates of corrosion and lower inhibition efficiencies at higher temperature in comparison to lower temperature may have been the result (Figure 10).

### 3.4 Adsorpttion isotherms

The adsorption isotherm study aims to illustrate the interfacial approach in the presence of the inhibitor, which is often a process of physical or chemical interaction between the substrate and adsorbate. The Bockris-Devanathan-Müller model states that the adsorption process in aqueous solution can be viewed as a substitution process between the organic compounds in the aqueous phase [Org (sol)] and water molecules at the electrode surface [H_2_O(ads)]. The recovery rate of adsorbate at the surface is crucial to achieving this (Eq. 5).

$$Org\left(sol\right)+xH_{2}O\left(ads\right)\to Org\left(ads\right)+xH_{2}O\left(sol\right)$$ (5)

Where x is the number of water molecules replaced by one organic inhibitor.

The adsorption isotherms provide a description of the inhibitor-metal surface interaction. Based on the weight loss results, the surface coverage (θ) values for various inhibitor concentrations in the seawater medium were calculated. The inhibitory efficiency is proportional to the fraction of the surface covered (θ) by adsorbed molecules. Eqs 6–11 illustrate how the adsorption process was described using the weight loss measurements that were collected from a variety of models (Langmuir, Freundlich, Temkin, Flory-Huggins, and El-Awady):

$$\begin{array}{l} El-Awady:\log\left(\frac{\theta }{1-\theta }\right)=\log k+y\log c \end{array}$$ (6)

$$Temkin:\theta =\left(-\frac{2.303}{2a}\right)\log k+\left(-\frac{2.303}{2a}\right)\log c$$ (7)

$$El-Awady:\log\left(\frac{\theta }{1-\theta }\right)=\log k+y\log c$$ (8)

$$Flory-Huggins:\log\left(\frac{c}{c}\right)=\log k+x\log\left(1-\theta \right)$$ (9)

$$Freundlich:\log\theta =\log k+\frac{1}{n}\log c$$ (10)

$$Langmuir:\frac{c}{\theta }=\frac{1}{k}+c$$ (11)

Where a is the lateral parameter of interaction between adsorbed molecules, c is the concentration, K is the adsorption constant, 1/n is the Freundlich exponent, x is the number of adsorbed water molecules substituted by inhibitor molecules, and y is the number of adsorbed molecules in an active site. The correlation coefficients for the Langmuir, Freundlich, and Temkin models for the examined isotherms were 0.966, 0.852, and 0.629, respectively.

**FIGURE 11:**
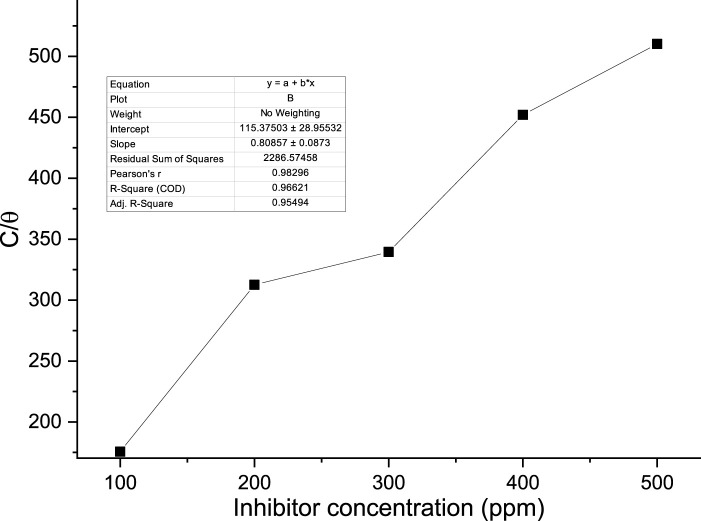
Langmuir adsorption isotherm of the PKSE extract.

The appropriate adsorption isotherm was found by graphically testing the data. The straight line (*R* ^2^ > 0.9) depicted in Figure 11 plot of C/θ against C indicates that adsorption follows the Langmuir adsorption isotherm. The Langmuir adsorption isotherm yielded the best fit. The adsorption characteristics for the Langmuir isotherm with an *R* ^2^ of 0.9662 are displayed in Table 6. According to the Langmuir isotherm, chemicals found in the palm kernel shell adsorb onto the metal surface.

**TABLE 6:** Adsorption parameters obtained with the PKSE extract on the corrosion inhibition of the steel.

Isotherm	*R* ^2^	y = a + bx
Langmuir	0.9662	y = 115.3 + 0.808x

### 3.5 Surface analysis

**FIGURE 12:**
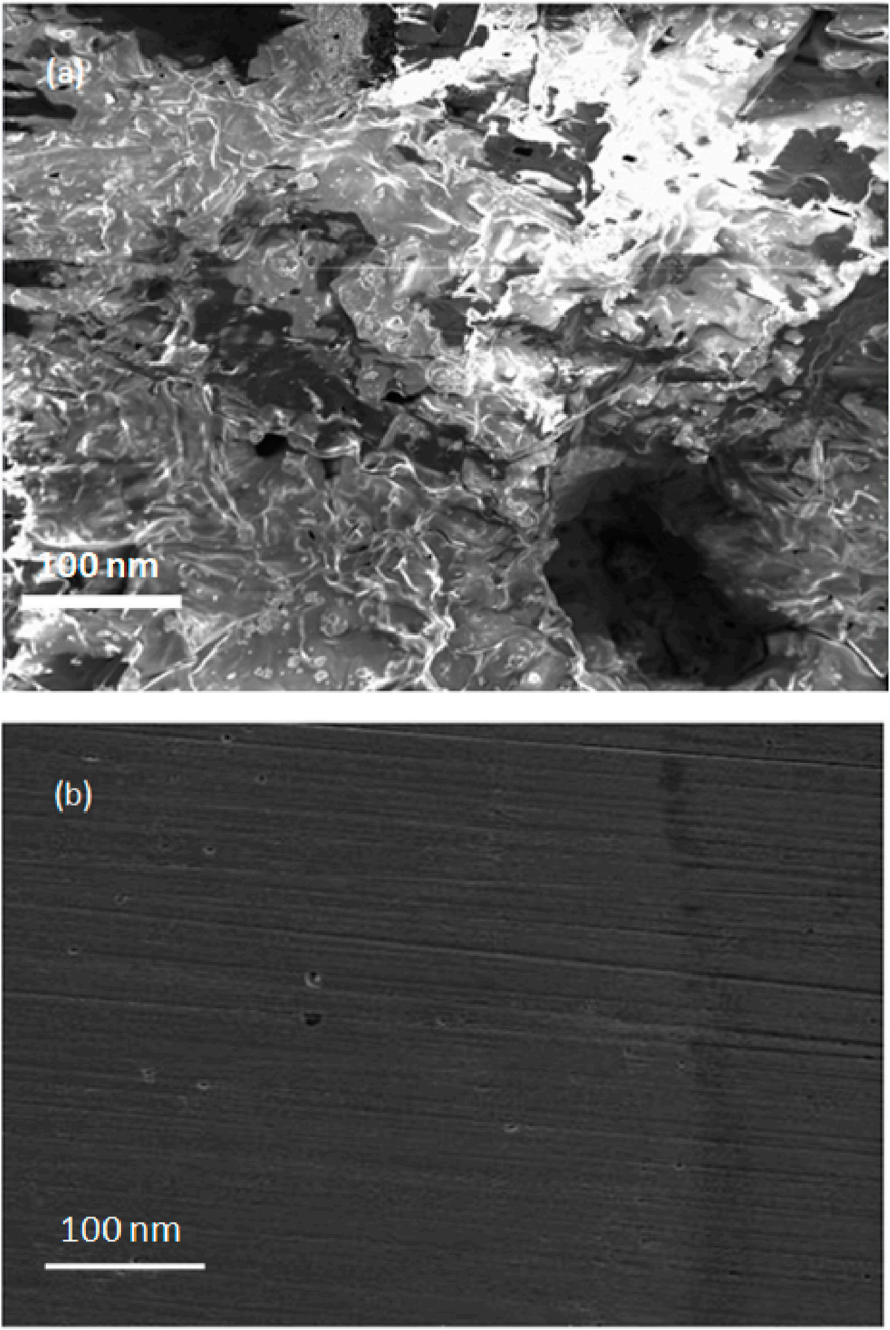
SEM images of the steel surface immersed in seawater **(A)** without **(B)** with 500 ppm PKSE extract.

The SEM analysis was carried out on the steel surface after gravimetric testing in the presence and absence of palm kernel shell extract (Figure 12). The SEM image for blank sample shows that the surface was significantly harmed by the saline media. The absence of corrosion assaults in the saline media containing the palm kernel shell, however, was observed, indicating that the steel surface was protected from corrosion by the thin inhibitor layers. Figure 12A SEM picture of damaged substrate demonstrates the porous corrosion products that result from extensive corrosion of steel in the aggressive saline solution. The steel substrate’s surface (Figure 12B) becomes smooth after PKSE is added to the saline solution, and a thin inhibitor film is clearly visible on it. This suggests that the inhibitor is present on the steel surface as an adsorbed layer that protects the metal and significantly slows down its rate of corrosion. The formation of an adsorbed molecular layer shields the metallic surface and lessens the severity of the corrosive process.

**FIGURE 13:**
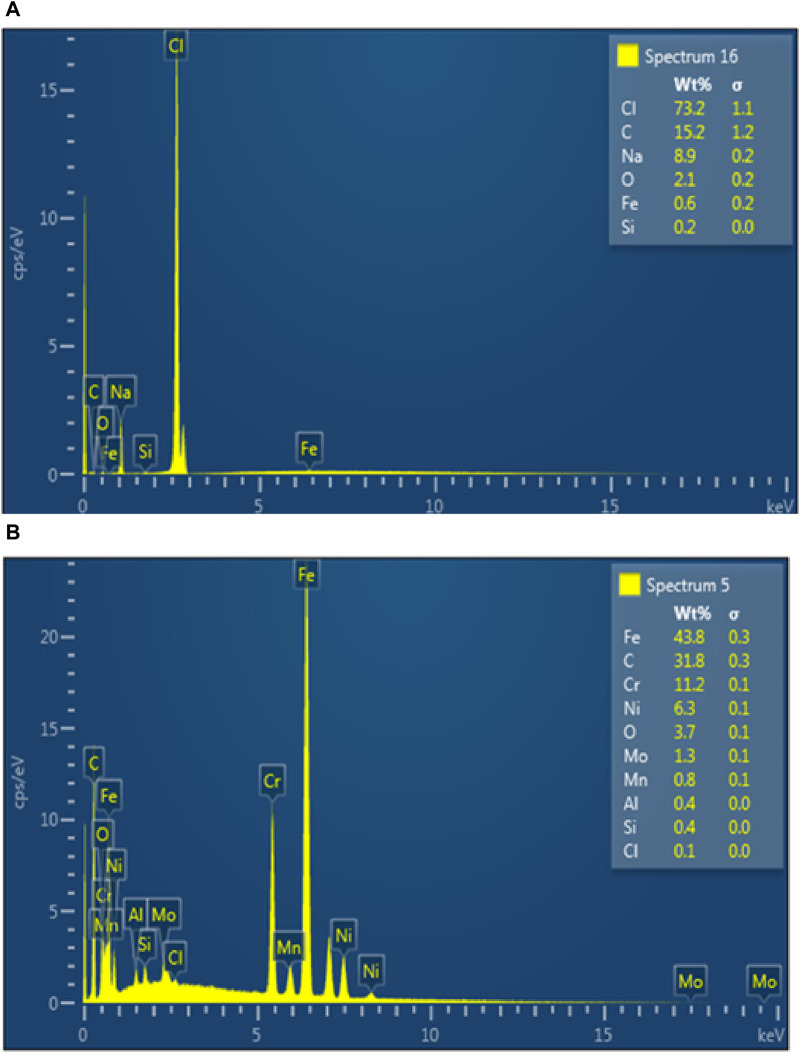
EDX spectra of the steel surface immersed in seawater **(A)** without **(B)** with 500 ppm PKSE extract.

To assess the chemical composition of the examined steel substrates, EDX studies (Figure 13) were performed. Although it is commonly known that EDX analysis cannot reliably determine the weight percentages of individual elements, it can be used to confirm the presence of elements with low atomic numbers. Na, Cl and Fe peaks were found in the blank saline solution (Figure 13A), indicating the development of corrosion products like iron oxides and hydroxides. On the other hand, C peak was seen in the resulting EDX graph with the addition of PKSE as corrosion inhibitors, confirming PKSE’s adsorption on the steel surface.

### 3.6 A comparison of the inhibition performance of palm kernel shell extract with previously studied extracts

Ecological friendliness with cost-effectiveness and inhibition performance is crucial factors that need to be taken into account at every step of inhibitor development as a result of the growing environmental consciousness. One of the reasons for the increased research activities is that, the majority of extracts from agricultural wastes are non-toxic. Table 7 presents a comparison between the inhibitory performance of palm kernel shell extract for steel in seawater and other extracts that were previously examined under comparable conditions. The investigated palm kernel shell extract exhibits inhibitory performance that is competitive with previous results. After 15 days at room temperature, 92% of the steel’s surface is protected with 500 ppm of palm kernel shell extract. It follows that extract from palm kernel shells can be used as an eco-friendly and efficient inhibitor that can be used in seawater environments.

**TABLE 7:** A comparison of inhibition performance of palm kernel shell extract for steel in seawater with previously studied extracts under similar conditions.

Inhibitor	Inhibition performance	References
Cashew nutshell extract	72%	Enabulele et al. (2023)
Egg shell extract	96.6	Kamaruzzaman et al. (2022)
Tobacco extract	83.9	Wang et al. (2016)
*Vicia faba* peel extracts	88.67–97.84	Abdelshafeek et al. (2022)
Sweet potato and turmeric extract	82.54%	Wijaya et al. (2023)
Egg shell extract	95.5%	Adeoye (2024)
Palm kernel shell extract	98%	This study

## 4 Conclusion

With the goal of eliminating harmful chemicals by the year 2030, corrosion experts are focusing more of their research on environmentally friendly alternatives. The palm kernel shell extract used in this work as a green corrosion inhibitor is made of natural materials that are easily obtained in the market; it is also biodegradable, and non-toxic. The palm kernel shell extract have demonstrated strong inhibitory performance in seawater solutions at various temperatures against the corrosion of thermo-mechanically treated steel. According to the gravimetric measurements, the extract’s inhibitory efficacy improves as its concentration in the reaction media increases. The solution’s temperature rise reduced the inhibitory effectiveness. 500 ppm is the optimum concentration, and at 303 K, the inhibition efficacy is 98%. The PKSE indicates that the extract influences both anodic and cathodic processes, indicating mixed type inhibitor, according to the polarization data. By using an adsorptive mechanism, the palm kernel shell extract suppresses and follows the Langmuir adsorption isotherms. The adsorption of the palm kernel shell extract on the thermo-mechanically treated steel surface is verified by the SEM and EDX analysis. An extract from palm kernel shells is an excellent inhibitor in seawater environments.

## Data Availability

The original contributions presented in the study are included in the article/Supplementary material, further inquiries can be directed to the corresponding author.
